# Clinical Characteristics of Systemic Lupus Erythematosus with Cirrhosis

**DOI:** 10.1155/2020/2156762

**Published:** 2020-05-21

**Authors:** Hanxiao You, Linyi Peng, Jiuliang Zhao, Yunyun Fei, Qian Wang, Wen Zhang, Mengtao Li, Xiaofeng Zeng

**Affiliations:** Department of Rheumatology, Peking Union Medical College Hospital, Peking Union Medical College & Chinese Academy of Medical Science, Key Laboratory of Rheumatology and Clinical Immunology, Ministry of Education, National Clinical Research Center for Dermatologic and Immunologic Diseases, Ministry of Science & Technology, No. 1 Shuaifuyuan, Beijing 100730, China

## Abstract

**Aim:**

Cirrhosis is rare in systemic lupus erythematosus (SLE) patients with a poor prognosis. This study is aimed at retrospectively analyzing our single-center experience to explore the characteristics of cirrhosis in SLE patients.

**Methods:**

SLE patients with cirrhosis from 2012 to 2019 were enrolled. SLE diagnosis was rigorously confirmed by a medical record review according to the revised 1997 American College of Rheumatology classification criteria for SLE. The diagnosis of liver cirrhosis was based on a combination of clinical, laboratory, and imaging criteria features. We conducted a case-control study in SLE patients complicated with the cirrhosis group and the age-, sex-, and entry-time-matched noncirrhosis group.

**Results:**

A total of 21 patients with SLE cirrhosis were enrolled, 3 males and 18 females. The median age at the time of cirrhosis diagnosis was 47.3 ± 4.0 years, and the mean disease duration of SLE before cirrhosis was 4.7 ± 1.0 years. The most common initial presentation was the involvement of the hematological system in 9 patients and then skin and mucosal involvement in 5 patients, arthritis in 4 patients, and nephritis in 3 patients. Patients with cirrhosis had a significantly higher rate of hematological system involvement (thrombocytopenia and leukopenia) and worse liver function; a higher level of immune globulin G had higher mortality (*p* < 0.05) than patients without cirrhosis.

**Conclusions:**

Cirrhosis is a rare and severe subtype of SLE with a poor prognosis. Those patients with hematological system involvement and impaired liver function should be paid more attention.

## 1. Introduction

The systemic lupus erythematosus (SLE) is an autoantibody-mediated, diffuse connective tissue disease with extremely variable and heterogeneous clinical presentation. A variety of organs can be involved, with the most common organ kidney, followed by the cardiovascular, nervous system, respiratory system, digestive system, blood system, etc., of which digestive damage, especially liver damage, is less common. The liver is not only a lymphoid organ involved in the immune response [1] but also a target of autoimmune reactions. The most common finding is an elevation in liver enzymes. Nevertheless, advanced liver disease with cirrhosis and liver failure is rare in patients with connective tissue diseases. The liver may be involved in 19.4% to 60% of patients with SLE at some point during the diseases, of which cirrhosis only accounts for about 1-2% [2–4]. Relatively, few studies have reported data of cirrhosis in SLE. We conduct this case-control study to explore the clinical characteristics of systemic lupus erythematosus with cirrhosis.

## 2. Patients and Methods

### 2.1. Study Population

This study was a single-center retrospective study. We utilized the Hospital Information Retrieval System to identify the SLE and cirrhosis patients admitted to the Peking Union Medical College Hospital (PUMCH) from January 2012 to December 2018. The records of each patient's hospitalization and outpatient visit can be checked by the patient's identification document (ID) in the medical record system. SLE diagnosis was rigorously confirmed by a medical record review according to the revised 1997 American College of Rheumatology classification criteria for SLE [5]. The diagnosis of liver cirrhosis was based on a combination of clinical, laboratory, and imaging criteria features (e.g., nodular liver, portosystemic collaterals found in abdominal echography, computerized tomography (CT), and/or magnetic resonance imaging (MRI)) [6]. We exclude other reason of cirrhosis such as alcoholic liver cirrhosis, nonalcoholic fatty liver disease, viral infection, drug-induced liver disease, inherited metabolic liver disease, cardiogenic liver disease, and other autoimmune liver disease, through screening medical history and systemic examination. For medical history, we focused on alcoholic abuse, hepatotoxic medication use, heart disease history, and family metabolic history. For systemic examination, we did a laboratory test such as hepatitis B/C virus test, ceruloplasmin test, autoantibody of primary biliary cholangitis (PBC) and autoimmune hepatitis (AIH), transthoracic echocardiograph, and hepatic vascular ultrasound. We present a case-controlled study, matched by age and gender, to discover the clinical characteristics of systemic lupus erythematosus with cirrhosis. The date of entry was the date of the first diagnosis of cirrhosis for the cirrhosis group and as the hospital admission date for the noncirrhosis group. Patients were followed until death, liver transplantation, or end of the study (July 2019). Written informed consent was obtained from each patient.

### 2.2. Clinical and Laboratory Data Collection

Medical records were retrospectively reviewed, and data were collected in a dedicated case report form. The following data were obtained from medical records by well-trained rheumatologists: gender, age at entry, duration of SLE, involved organs, lupus disease activity, and laboratory data. Lupus disease activity was evaluated using the SLE disease activity index 2000 (SLEDAI-2K), stratified to stable (<5), mild active (5-9), moderate active (10-14), and severe active (>14). The severity of liver diseases was evaluated with the Child-Pugh score. Clinical ascites, hepatic encephalopathy, and the other complications were classified as absent or present. Routine blood tests, including platelet count, hemoglobin, liver and renal function tests, and prothrombin time, were collected. All parameters used for comparison were those at the time of diagnosis of cirrhosis for the cirrhosis group and the diagnosis of SLE for the noncirrhosis group.

### 2.3. Antibody Assay

The anti-nuclear antibody (ANA) was tested by indirect immunofluorescence (IIF) using HEp-2 cell substrates. The anti-dsDNA antibody was measured by IIF using flagellate protoctista substrates and enzyme-linked immunosorbent assay (ELISA). Anti-ENA antibodies were determined by immunodiffusion assay. IgG/IgM antibody of ACL and anti-*β*2-glycoprotein I were tested by ELISA. LAC was measured using activated partial thromboplastin time- (APTT-) based assay.

### 2.4. Statistical Analysis

Continuous variables were presented as means and standard deviation (SD) for normally distributed data, and medians and interquartile range (IQR) for all other data, whereas categorical variables were presented as number (percentage). Continuous variables were compared with the use of Student's *t* test or the Mann-Whitney test, while categorical variables were compared using the chi-square test or Fisher's exact test. The *p* value was two tailed and defined as significant if the value was <0.05. SPSS software, version 23 (Chicago, IL, USA) was used for all of the statistical descriptions, analyses, and inferences.

## 3. Results

Among 6994 SLE inpatients in our center between 2012 and 2018, a total of 24 SLE patients with cirrhosis were enrolled; then, 3 patients with PBC or AIH were excluded. Finally, 21 patients were included as the cirrhosis group, 18 females and 3 males. The median age at the time of cirrhosis diagnosis was 47.3 ± 4.0 years, and the mean disease duration of SLE before cirrhosis was 4.7 ± 1.0 years. There was no liver transplant, and 5 patients died in the cirrhosis group. One patient died of hepatic encephalopathy, two of gastrointestinal bleeding, and two of pulmonary infection. Twenty-one contemporaneous patients hospitalized in our center were randomly selected to create the control cohorts of the SLE patients without cirrhosis.

We analyzed the characteristics of the cirrhosis group (Table 1). Among 21 SLE patients with cirrhosis, the most common initial presentation was the involvement of the hematological system in 9 patients and then skin and mucosa involvement in 5 patients, arthritis in 4 patients, and nephritis in 3 patients. As for cirrhotic symptoms, 9 patients had the symptom of increased abdominal girth and 12 patients of edema. As for complications, 3 patients had gastrointestinal bleeding, 11 patients had hypersplenism, 5 patients had hepatic encephalopathy, 13 patients had portal hypertension, 9 patients had esophageal and gastric varices, 13 patients had ascites, and 2 patients had portal vein thrombosis (PVT). Child-Pugh score: A 1 (5%) patient, B 14 (67%) patients, and C 6 (29%) patients.

Comparison of demographic characteristics, clinical and laboratory manifestations between cirrhosis and noncirrhosis groups in SLE patients are shown in Tables 2 and 3. There was no significant difference in gender and age at study entry between cirrhosis and noncirrhosis groups. The rate of thrombocytopenia (81%), hypocomplementemia (95%), pulmonary arterial hypertension (PAH) (33%), thrombus (29%), and death (24%) was significantly higher in the cirrhosis group (*p* < 0.05). Low serum levels of albumin (ALB), prealbumin (PA), and creatinine (Cr) and high levels of alanine transaminase (ALT), glutamic oxalacetic transaminase (AST), total bilirubin (TBIL), direct bilirubin (DBIL), gamma-glutamyl transpeptidase (GGT), alkaline phosphatase (ALP), and immune globulin (Ig) were found in cirrhotic patients.

The comparison of antibodies between cirrhosis and noncirrhosis groups is shown in Table 4. Serologic tests showed that all patients were positive for antinuclear antibody (ANA) and 14 patients were positive for double-stranded DNA antibody, and no differences were found between patients with and without cirrhosis.

As for the treatment (Table 2), 16 patients in the cirrhosis group and 16 patients in the noncirrhosis group were treated with a high dose of glucocorticoid initially (1 mg/kg/day) and tapered down at a regular interval. There is no significant difference in the initial dosage of glucocorticoid between the cirrhosis group and the noncirrhosis group (50 (30, 60) vs. 55 (42, 60), *p* = 0.383). The use of cyclophosphamide (CYC) in the cirrhosis group was significantly lower than that in the noncirrhosis group (*p* = 0.009); otherwise, immunosuppressants had no difference between the two groups, including mycophenolate mofetil (MMF), tacrolimus (FK506), and hydroxychloroquine (HCQ). In the cirrhosis group, symptomatic treatment included diuretic treatment (*n* = 18), albumin infusion (*n* = 11), and catharsis of the gut (*n* = 7).

## 4. Discussion

The liver is the largest lymphoid organ involved in the immune response and in the maintenance of tolerance to self molecules [6]. However, it is also a target of autoimmune reactions, as observed in primary autoimmune liver diseases (AILD) such as AIH, PBC, and primary sclerosing cholangitis (PSC). Further, the liver is frequently affected by connective tissue diseases (CTD), including systemic lupus erythematosus, antiphospholipid syndrome, primary Sjogren's syndrome, systemic sclerosis, vasculitis, dermatomyositis, polymyositis, and antisynthetase syndrome, most commonly in the form of abnormal liver function test with predominant cholestatic or hepatocellular damage [2, 3, 7–10]. Although liver cirrhosis and failure are extremely rare in patients with SLE, unusual liver conditions such as nodular regenerative hyperplasia or Budd-Chiari syndrome have been reported with increasing frequency in patients with CTD [8]. Lupus hepatitis can occur at any stage of the disease, and the pathologic histology only showed mild-to-moderate nonspecific changes, such as liver steatosis, inflammatory cell infiltration in the portal area, and a few inflammations in the small blood vessels in the liver. The deposition of complement and immune complex in the vascular wall and the lumen partial or complete occlusion leads to liver cell necrosis and regeneration, the formation of nodules, and eventually develop into cirrhosis of the liver [11, 12].

The most common initial manifestation of SLE cirrhosis was the involvement of the hematological system. The rate of the involved hematological system, including thrombocytopenia and leukopenia, is also significantly higher in cirrhosis patients. Systemic lupus erythematosus itself can involve the hematological system. Hypersplenism due to cirrhosis may also contribute to the peripheral cytopenia [13].

In our study, 20 patients (95%) had Child-Pugh class B or C, and 18 patients (86%) had at least one liver-related complication. When compared with the noncirrhosis group, SLE patients with cirrhosis had a significantly higher rate of death, and more than half of patients' deaths were attributed to cirrhosis, suggesting a worse prognosis than those SLE patients without cirrhosis. The rate of PAH is also higher in the cirrhosis group. Portopulmonary hypertension (PPHT) is a respiratory complication of portal hypertension and is the 4th subtype in frequency in the group of pulmonary arterial hypertension [14]. The pathogenesis of PPHT may result from an imbalance between vasoconstrictor and vasodilating factors [15]. The presence of both portal and pulmonary vascular disease contributes to complicated hemodynamics and therapeutic challenges [16].

According to the laboratory analysis, the liver function is significantly poor in cirrhosis patients than that in the noncirrhosis group. The liver is not usually a major target organ for damage, and abnormal liver function is not included in the classification and diagnostic criteria for SLE. However, liver dysfunction is common in SLE and is found in up to 60% of patients at a certain point in their illness [2–4], mainly due to disease activity and drug toxicity and only rarely an overlapping primitive autoimmune liver disease. Almost all drugs in the armamentarium against SLE or other rheumatologic diseases may cause liver toxicity, as represented by nonsteroidal anti-inflammatory drugs (NSAID). Liver damage can also occur in the overlap syndrome of SS, APS, and AILD with SLE. The reported prevalence of AIH or PBC overlap syndrome is similar in SLE patients (2.7%), and a family history of SLE is independently associated with PBC in a large case-control study [10]. Both SLE-associated hepatitis and AIH manifest with polyarthralgia, elevated gamma globulins, and a positive antinuclear antibody creating a diagnostic dilemma. It is important to differentiate between SLE-associated hepatitis and AIH because of therapeutic and prognostic implications. In some cases, it may be difficult to distinguish between AIH and SLE-associated hepatitis. Serum antibodies, complement levels, and liver histology may thus be of help. SS can be secondary to SLE. Liver diseases associated with SS include PBC, AIH, viral hepatitis (B and C), sclerosing cholangitis, NRH, and cirrhosis [7]. Antiphospholipid syndrome, an autoimmune disorder that is commonly found with SLE and manifests with arterial and venous thrombosis, is associated with Budd-Chiari syndrome [17].

Antibodies to ribosomal P protein have been shown to be strongly correlated with lupus hepatitis, being detected in a significant proportion of patients (69%) [18]. However, there is no difference in the antibodies between the two groups in our study. The possible reason for this result may be the small sample size. In our study, the use of CYC in the cirrhosis group was significantly lower than that in the noncirrhosis group. The reason may be that patients with cirrhosis are more likely to have abnormal routine blood tests and abnormal liver function [19].

Our research had several limitations. The major limitation of the study is the small sample size. Considering the low incidence of SLE associated, longer follow-up is required. Secondly, we did not obtain the pathological results of the patient's liver biopsy. Liver biopsy has been the gold standard for the diagnosis of liver cirrhosis [6]. However, no liver biopsy was performed in the cirrhosis group because of contraindications, including thrombocytopenia (*n* = 10), severe coagulopathy (*n* = 5), or large amount of ascites (*n* = 15). Moreover, liver biopsy was an invasive procedure and cirrhosis can be diagnosed clinically. In general, we still need more studies to explore the characteristics and mechanism of cirrhosis in SLE patients, to improve the prognosis of these patients.

## 5. Conclusions

In conclusion, cirrhosis is a rare and severe subtype of SLE with a poor prognosis. Those patients with hematological system involvement and impaired liver function should be paid more attention. Further studies with larger samples are needed to deepen the understanding of the risk of cirrhosis in SLE patients, achieve early identification and timely intervention, and ultimately improve the prognosis of those patients.

## Figures and Tables

**Table 1:** The characteristics at baseline of the cirrhosis group.

The initial manifestation of SLE	
Skin and mucosa, *n* (%)	5 (24%)
Arthritis, *n* (%)	4 (19%)
Hematological system, *n* (%)	9 (43%)
Nephritis, *n* (%)	3 (14%)
Cirrhotic symptoms	
Abdominal pain, *n* (%)	1 (5%)
Increased abdominal girth, *n* (%)	9 (43%)
Edema, *n* (%)	12 (57%)
Jaundice, *n* (%)	2 (10%)
Complications	
GIB, *n* (%)	3 (14%)
Hypersplenism, *n* (%)	11 (52%)
Hepatic encephalopathy, *n* (%)	5 (24%)
Portal hypertension, *n* (%)	13 (62%)
Esophageal and gastric varices, *n* (%)	9 (43%)
Ascites, *n* (%)	13 (62%)
PVT, *n* (%)	2 (10%)
Child-Pugh score	
A, *n* (%)	1 (5%)
B, *n* (%)	14 (67%)
C, *n* (%)	6 (29%)

SLE: systemic lupus erythematosus; GIB: gastrointestinal bleeding; PVT: portal venous thrombosis.

**Table 2:** Comparison of demographic characteristics and clinical manifestations between cirrhosis and noncirrhosis groups in SLE patients.

Characteristics of patients	Cirrhosis group (*n* = 21)	Noncirrhosis group (*n* = 21)	*p* value
Demographics			
Sex (female), *n* (%)	18 (87.5%)	18 (87.5%)	1
Age (years), mean ± SD	47.3 ± 4.0	45.9 ± 3.6	0.79
Disease duration (years), mean ± SD	4.7 ± 1.0	6.4 ± 1.7	0.392
Complications			
Diabetes, *n* (%)	1 (5%)	3 (14%)	0.606
High blood pressure, *n* (%)	7 (33%)	10 (48%)	0.346
Hepatitis B, *n* (%)	1 (5%)	2 (10%)	1
sSS, *n* (%)	3 (14%)	3 (14%)	1
sAPS, *n* (%)	6 (29%)	2 (10%)	0.238
Clinical manifestations			
Rash, *n* (%)	10 (48%)	8 (38%)	0.533
Leukopenia, *n* (%)	12 (57%)	5 (24%)	0.028^∗^
Anemia, *n* (%)	15 (71%)	15 (71%)	1
Thrombocytopenia, *n* (%)	17 (81%)	7 (33%)	0.004^∗^
Nervous system, *n* (%)	4 (19%)	6 (29%)	0.719
Nephritis, *n* (%)	9 (43%)	13 (62%)	0.217
Serositis, *n* (%)	2 (10%)	5 (24%)	0.41
Arthritis, *n* (%)	7 (33%)	6 (29%)	0.739
Interstitial lung disease, *n* (%)	3 (14%)	4 (19%)	1
PAH, *n* (%)	7 (33%)	1 (5%)	0.045^∗^
Thrombus, *n* (%)	6 (29%)	2 (10%)	0.021^∗^
SLE disease activity index			
SLEDAI, median (IQR)	6 (4, 12)	7 (0, 15.5)	0.913
SLEDAI (0-4), *n* (%)	6 (29%)	6 (29%)	1
SLEDAI (5-9), *n* (%)	5 (24%)	7 (33%)	0.734
SLEDAI (10-14), *n* (%)	5 (24%)	2 (10%)	0.41
SLEDAI (>14), *n* (%)	3 (24%)	6 (29%)	0.454
Treatment			
Initial glucocorticoid (mg/d), median (IQR)	50 (30, 60)	55 (42, 60)	0.383
Initial immunosuppressive agents			
CYC	3 (24%)	12 (57%)	0.009^∗^
MMF	5 (24%)	2 (10%)	0.410
FK506	4 (19%)	1 (5%)	0.343
HCQ	15 (71%)	16 (76%)	0.726
Prognosis			
Death, *n* (%)	5 (24%)	0 (0%)	0.048^∗^

SD: standard deviation; IQR: interquartile range; SLEDAI: SLE disease activity index; PAH: pulmonary arterial hypertension; sSS: secondary Sjogren's syndrome; sAPS: secondary antiphospholipid syndrome; CYC: cyclophosphamide; MMF: mycophenolate mofetil; FK506: tacrolimus; HCQ: hydroxychloroquine. ^∗^*p* < 0.05.

**Table 3:** Comparison of laboratory index between cirrhosis and noncirrhosis groups in SLE patients.

Laboratory variables	Cirrhosis group (*n* = 21)	Noncirrhosis group (*n* = 21)	*p* value
HGB (g/L), mean ± SD	91.1 ± 4.82	102.19 ± 5.88	0.15
WBC (/*μ*L), mean ± SD	4.53 ± 0.83	6.63 ± 0.83	0.08
PLT (/*μ*L), mean ± SD	77.62 ± 13.78	188.24 ± 20.71	<0.01^∗^
Cr (*μ*mol/L), mean ± SD	69.99 ± 7.46	124.1 ± 24.72	0.04^∗^
BUN (mmol/L), mean ± SD	8.49 ± 0.96	12 ± 1.99	0.13
ALB (g/L), mean ± SD	27.1 ± 1.35	33.19 ± 1.32	<0.01^∗^
ALT (U/L), mean ± SD	56.43 ± 9.46	18.48 ± 2.63	<0.01^∗^
AST (U/L), mean ± SD	67.43 ± 11.76	22.38 ± 3.9	<0.01^∗^
TBIL (*μ*mol/L), mean ± SD	37.96 ± 12.24	11.34 ± 2.67	0.04^∗^
DBIL (*μ*mol/L), mean ± SD	20.87 ± 9.32	5.2 ± 2.2	0.11
GGT (U/L), mean ± SD	97.43 ± 15.2	45.5 ± 10.53	0.01^∗^
ALP (U/L), mean ± SD	131.81 ± 17.54	64.7 ± 6.33	<0.01^∗^
LDH (U/L), mean ± SD	248.33 ± 24.37	281.45 ± 37.28	0.46
CHE (U/L), mean ± SD	3.08 ± 0.38	5.27 ± 0.43	<0.01^∗^
PA (mg/L), mean ± SD	151.95 ± 22.32	267.8 ± 20.15	<0.01^∗^
IgG (g/L), mean ± SD	27.25 ± 2.86	11.63 ± 1.17	<0.01^∗^
IgA (g/L), mean ± SD	4.84 ± 0.7	2.63 ± 0.32	0.01^∗^
IgM (g/L), mean ± SD	1.88 ± 0.22	0.95 ± 0.16	<0.01^∗^
Fbg (g/L), mean ± SD	2.42 ± 0.23	2.73 ± 0.27	0.39
hsCRP (mg/L), mean ± SD	26.8 ± 7.28	9.48 ± 4.44	0.05
ESR (mm/1 h), mean ± SD	62.44 ± 8.66	9.48 ± 4.44	<0.01^∗^
Hypocomplementemia, *n* (%)	20 (95%)	12 (57%)	0.009^∗^

SD: standard deviation; WBC: white blood cell; PLT: platelet; BUN: urea nitrogen; ALB: albumin; ALT: alanine transaminase; AST: glutamic oxaloacetic transaminase; TBIL: total DBIL, direct bilirubin; GGT: gamma-glutamyl transpeptidase; ALP: alkaline phosphatase; LDH: lactate dehydrogenase; CHE: cholinesterase; PA: prealbumin; hsCRP: hypersensitive c-reactive protein; ESR: erythrocyte sedimentation rate; ^∗^*p* < 0.05.

**Table 4:** Comparison of antibodies between cirrhosis and noncirrhosis groups in SLE patients.

Autoantibodies (positive)	Cirrhosis group (*n* = 21)	Noncirrhosis group (*n* = 21)	*p* value
ANA, *n* (%)	21 (100%)	21 (100%)	1
anti-dsDNA, *n* (%)	14 (67%)	12 (57%)	0.53
anti-Sm, *n* (%)	5 (24%)	5 (24%)	1
anti-RNP, *n* (%)	7 (33%)	4 (19%)	0.29
AUNA, *n* (%)	4 (19%)	4 (19%)	1
anti-SSA, *n* (%)	12 (57%)	8 (38%)	0.22
anti-SSB, *n* (%)	3 (14%)	3 (14%)	1
anti-Ro52, *n* (%)	4 (19%)	7 (33%)	0.29
anti-rRNP, *n* (%)	2 (10%)	2 (10%)	1
AHA, *n* (%)	5 (24%)	5 (24%)	1
ACA, *n* (%)	3 (14%)	0 (0%)	0.12
ACL, *n* (%)	4 (19%)	0 (0%)	0.11
anti*β*2GPI, *n* (%)	4 (19%)	1 (5%)	0.34
LA, *n* (%)	2 (10%)	2 (10%)	1
Coombs test, *n* (%)	10 (48%)	4 (19%)	0.05

ANA: anti-nuclear antibodies; anti-dsDNA: anti-double-stranded DNA; anti-Sm: anti-Smith; anti-RNP: antiribonucleoprotein; AUNA: anti-nucleosome antibodies; anti-SSA: anti-SSA/Ro; anti-SSB: anti-SSB/La; anti-rRNP: antiribosomal RNP; AHA: anti-histone antibody; AMA: antimitochondrial antibodies; ACA: anti-centromere antibody; ACL: anticardiolipin; anti*β*2GPI: anti-*β*2-glycoprotein I; LA: lupus anticoagulant.

## Data Availability

The data used to support the findings of this study are available from the corresponding authors upon request.

## Abbreviations

SLE: Systemic lupus erythematosus
GIB: Gastrointestinal bleeding
PVT: Portal venous thrombosis
PBC: Primary biliary cholangitis
AIH: Autoimmune hepatitis
SD: Standard deviation
IQR: Interquartile range
SLEDAI: SLE disease activity index
PAH: Pulmonary arterial hypertension
sSS: Secondary Sjogren's syndrome
sAPS: Secondary antiphospholipid syndrome
WBC: White blood cell
PLT: Platelet
BUN: Urea nitrogen
ALB: Albumin
ALT: Alanine transaminase
AST: Glutamic oxaloacetic transaminase
TBIL: Total DBIL, direct bilirubin
GGT: Gamma-glutamyl transpeptidase
ALP: Alkaline phosphatase
LDH: Lactate dehydrogenase
CHE: Cholinesterase
PA: Prealbumin
hsCRP: Hypersensitive c-reactive protein
ESR: Erythrocyte sedimentation rate
ANA: Anti-nuclear antibodies
anti-dsDNA: Anti-double-stranded DNA
anti-Sm: Anti-Smith
anti-RNP: Antiribonucleoprotein
AUNA: Anti-nucleosome antibodies
anti-SSA: Anti-SSA/Ro
anti-SSB: Anti-SSB/La
anti-rRNP: Antiribosomal RNP
AHA: Anti-histone antibody
AMA: Antimitochondrial antibodies
ACA: Anti-centromere antibody
ACL: Anticardiolipin
anti*β*2GPI: Anti-*β*2-glycoprotein I
LA: Lupus anticoagulant
CYC: Cyclophosphamide
MMF: Mycophenolate mofetil
FK506: Tacrolimus
HCQ: Hydroxychloroquine.

## References

[1] Selmi C., Mackay I., Gershwin M. (2007). The immunological milieu of the liver. *Seminars in Liver Disease*.

[2] Youssef W. I., Tavill A. S. (2002). Connective tissue diseases and the liver. *Journal of Clinical Gastroenterology*.

[3] Takahashi A., Abe K., Saito R. (2013). Liver dysfunction in patients with systemic lupus erythematosus. *Internal Medicine*.

[4] Efe C., Purnak T., Ozaslan E. (2011). Autoimmune liver disease in patients with systemic lupus erythematosus: a retrospective analysis of 147 cases. *Scandinavian Journal of Gastroenterology*.

[5] Hochberg M. C. (1997). Updating the American College of Rheumatology revised criteria for the classification of systemic lupus erythematosus. *Arthritis & Rheumatism*.

[6] Fukui H., Saito H., Ueno Y. (2016). Evidence-based clinical practice guidelines for liver cirrhosis 2015. *Journal of Gastroenterology*.

[7] Selmi C., de Santis M., Gershwin M. E. (2011). Liver involvement in subjects with rheumatic disease. *Arthritis Research & Therapy*.

[8] De Santis M., Crotti C., Selmi C. (2013). Liver abnormalities in connective tissue diseases. *Best Practice & Research. Clinical Gastroenterology*.

[9] Gebreselassie A., Aduli F., Howell C. D. (2019). Rheumatologic diseases and the liver. *Clinics in Liver Disease*.

[10] Efe C., Ozaslan E., Nasiroglu N., Tunca H., Purnak T., Altiparmak E. (2010). The development of autoimmune hepatitis and primary biliary cirrhosis overlap syndrome during the course of connective tissue diseases: report of three cases and review of the literature. *Digestive Diseases and Sciences*.

[11] Matsumoto T., Yoshimine T., Shimouchi K. (1992). The liver in systemic lupus erythematosus: pathologic analysis of 52 cases and review of Japanese autopsy registry data. *Human Pathology*.

[12] Laxer R. M., Roberts E. A., Gross K. R. (1990). Liver disease in neonatal lupus erythematosus. *The Journal of Pediatrics*.

[13] Lv Y., Yee Lau W., Wu H. (2017). Causes of peripheral cytopenia in hepatitic cirrhosis and portal hypertensive splenomegaly. *Experimental Biology and Medicine*.

[14] Rodríguez-Almendros N., Toapanta-Yanchapaxi L. N., Valadez J. A., Zavaleta N. E., Muñoz-Martínez S. G., García-Juárez I. (2018). Hipertensión portopulmonar: revisión actualizada. *Archivos de Cardiología de México*.

[15] Hopps E., Valenti A., Caimi G. (2011). Portopulmonary hypertension. *Clinical and Investigative Medicine*.

[16] Fussner L. A., Krowka M. J. (2016). Current approach to the diagnosis and management of portopulmonary hypertension. *Current Gastroenterology Reports*.

[17] Uthman I., Khamashta M. (2007). The abdominal manifestations of the antiphospholipid syndrome. *Rheumatology*.

[18] Runyon B. A., LaBrecque D. R., Anuras S. (1980). The spectrum of liver disease in systemic lupus erythematosus. *The American Journal of Medicine*.

[19] Angeli P., Bernardi M., Villanueva C. (2018). EASL clinical practice guidelines for the management of patients with decompensated cirrhosis. *Journal of Hepatology*.

